# Effect of Reinforcements and 3-D Printing Parameters on the Microstructure and Mechanical Properties of Acrylonitrile Butadiene Styrene (ABS) Polymer Composites

**DOI:** 10.3390/polym14102105

**Published:** 2022-05-21

**Authors:** Ved S. Vakharia, Mrityunjay Singh, Anton Salem, Michael C. Halbig, Jonathan A. Salem

**Affiliations:** 1 NASA Pathway Intern, Department of Mechanical and Aerospace Engineering, University of California, San Diego, CA 92092, USA; ved.vakharia@gmail.com; 2 Ohio Aerospace Institute, Cleveland, OH 44142, USA; 3 NASA Intern Currently at VulcanForms, Inc., Burlington, MA 01803, USA; antonsalem1@gmail.com; 4 NASA Glenn Research Center, Cleveland, OH 44135, USA; jonathan.a.salem@nasa.gov

**Keywords:** Acrylonitrile Butadiene Styrene (ABS), 3-D printing, polymer composites, multifunctionality, fused filament fabrication, carbon-reinforced ABS, mechanical properties

## Abstract

Fused filament fabrication (FFF) systems utilize a wide variety of commercially available filaments, including Acrylonitrile Butadiene Styrene (ABS), as well as their variants. However, the effect of filament composition, reinforcements (chopped fibers and nanotubes), and 3-D printing variables on the microstructure and thermomechanical behavior is not well understood, and systematic studies are needed. In this work, different types of ABS materials with and without carbon fiber and carbon nanotube reinforcements were printed with multiple print layer heights. The microstructure, elastic behavior, tensile behavior, and fracture toughness of 3-D printed materials were characterized. ABS material systems printed at a low print layer height of 0.1 mm outperformed those printed at a larger height of 0.2 mm. Carbon nanotube reinforcements result in significant improvement in the strength and elastic modulus of ABS materials. Printed coupons of ABS with carbon nanotubes achieve an ultimate strength of 34.18 MPa, while a premium grade ABS coupon achieved 28.75 MPa when printed with the same print layer heights. Samples of ABS with chopped carbon fiber show an ultimate strength of 27.25 MPa, due primarily to the significant porosity present in the filament. Elastic moduli and fracture toughness measured using dynamic and mechanical methods show similar trends as a function of layer height. The effects of different materials, reinforcements, and printing parameters on the microstructure and mechanical properties are discussed in detail.

## 1. Introduction

In recent years, there has been a growing demand for new materials and manufacturing processes for lightweight multifunctional components and systems. New materials are being explored to meet new performance requirements. Technological and material advances have led to significant improvements in various industries such as automotive, aerospace, energy, and medical. In general, traditional manufacturing techniques like machining are subtractive. However, additive manufacturing (AM) forms objects layer by layer by joining interlayers. In the last several years, significant advances have been made in the development of a wide array of 3-D printing systems ranging from commercially available high-end systems for fused filament fabrication (FFF), also referred to as fused deposition modeling (FDM), and affordable desktop 3-D printers or polymer system. 3-D printing holds multiple advantages over traditional manufacturing methods. 3-D printing technologies can produce far more complex geometries while also eliminating the need for tool production. Furthermore, 3-D printers are incredibly versatile and can print with varying materials and parameters, allowing for the introduction of multifunctionality by integrating conductive additives to the host polymer material. For example, there is interest in applying rapid manufacturing to FDM 3D printing for applications as ambitious as prosthetics and other bionic or soft-robotic applications [1].

Since most desktop low-cost printers and their RepRap variants can fabricate materials from acrylonitrile butadiene styrene (ABS) and polylactic acid (PLA) systems, there is much interest in these materials. In previous studies, the Fused Filament Fabrication (FFF) processing and properties of ABS materials using commercial machines have been reported [2,3,4]. Recently, Tymrak et al. [5] have measured the tensile strength and elastic modulus of printed components using a selection of open-source 3-D printers. Their results find average tensile strengths of 28.5 MPa for ABS and 56.6 MPa for PLA with average elastic moduli of 1807 MPa for ABS and 3368 MPa for PLA. Life cycle analysis [6,7] results suggest a potential lower environmental impact of distributed manufacturing using 3D printers rather than conventional manufacturing for various products.

It is important to note that, traditionally, ABS and PLA were the two most common polymeric materials used in 3-D printing. However, a more recent interest in 3D printing, specifically FDM, has arisen because of the manufacturing method’s ability to easily incorporate nanoparticles or other reinforcements [8,9,10,11]. In recent years, composite materials have emerged, such as carbon-fiber-reinforced ABS or even carbon nanotube reinforced ABS. The cost of these materials is highly variable, ranging from rather expensive industrial-grade materials to inexpensive pellets (1$/lb.) that can be used to create filament “in-house”.

Studies have shown significant improvements in tensile modulus and strength in 3D-printed ABS material systems reinforced with carbon nanotubes [12] and chopped carbon fiber [13,14]. For example, Dul et al. [8] found the addition of carbon nanotubes to increase the tensile strength of 3D-printed ABS from 45.7 MPa to 49.6 MPa, and Love et al. [14] demonstrated a yield strength of 70 MPa in ABS materials with 15 wt.% chopped carbon fiber. However, there is an informational void on print layer height’s effects on the microstructure and mechanical properties of 3D-printed ABS material systems. Dul et al. [8] did acknowledge the presence of voids in 3D-printed ABS with carbon nanotubes, which will be investigated in this study, and Tekinalp et al. [15] did find that as more chopped carbon fiber was introduced to the ABS filament, more porosity was observed in the 3D-printed specimens. One parameter controlled in FFF is the print layer height, thereby controlling the print speed. With larger print layer heights, for example, 0.3 mm compared to 0.1 mm, 3D-printed parts can be completed in a shorter time. Changes in the microstructure and porosity that occur with increasing print layer heights have not been studied. A larger print layer height, and therefore a faster print, may be detrimental to the mechanical integrity of the part. Reinforcements such as carbon nanotubes and chopped fiber may play a part in preventing or exacerbating these detrimental effects. As was conducted for PLA systems with additions [16], these relations in ABS material systems are investigated in this study.

Various ABS materials from different sources were printed using desktop 3-D printers in this study. Microstructure, density, tensile strength, fracture toughness, and elastic modulus of these materials were evaluated as a function of printing parameters. Specimens were printed with 0.1 mm, 0.2 mm, 0.3 mm, and 0.4 mm layer heights. This research aimed to determine how different starting materials and printing parameters affect mechanical properties. The effects of the layer height of printed materials and the presence of carbon fiber and nanotube reinforcements on the mechanical properties were evaluated.

## 2. Materials and Methods

Makerbot Replicator 2X (MakerBot Industries, LLC One MetroTech Center, Brooklyn, NY 11201, USA) and Orion Delta 3-D (SeeMeCNC, Ligonier, IN 46767, USA) printers were used in this work to print ASTM D638 [17] tensile and ASTM D5045 [18] fracture toughness specimens. These specimens were printed with 0.1 mm, 0.2 mm, 0.3 mm, and 0.4 mm layer heights. Parts were printed at alternating raster angles of 0° and 45°. The final shapes of the parts were made according to the aforementioned ASTM standards for tensile and fracture testing. In addition to varying the layer height of the printed specimens, the print material was changed. Different materials used in this study were multi-walled carbon nanotube reinforced ABS (3DXTech), ABS made in-house from pure ABS pellets (Filabot (Filabot, Barre, VT 05641, USA)), “premium” grade ABS (3DXTech), 5 wt.% carbon fiber reinforced ABS (3DXTech), and ABS Black (3DXTech), and will be hereby referred to as ABS w/CNT, Lab-made ABS, Premium ABS, ABS w/5%CF, and Pure ABS, respectively. Three tensile coupons and three fracture toughness specimens of each layer type and material type were 3D printed and then tested, except for the case of ABS w/5%CF and Pure ABS in which only 1 fracture toughness specimen per layer height was produced. Lab-made ABS was made from short filaments produced from Filabot ABS pellets by melt extrusion in a Filabot extruder. This process was performed on a small scale, relative to the other ABS materials sourced from external manufacturers. The Lab-made ABS had limited testing as a result.

Thermogravimetric analysis (TGA) was performed with a TGA Q500 Thermogravimetric analyzer (TA Instruments (TA Instruments, New Castle, DE 19720, USA)) for bulk filament pieces to not only estimate the wt.% addition of reinforcement, but also to observe differences in impurities present in the filaments. The instrument was calibrated for temperature, heat flow, and weight according to the manufacturer’s suggestions. Specimens were cut from filament and tested. TGA scans were run from room temperature (25 °C) to 745 °C at the heating rate of 10 °C/min. The sample purge was 60 cc/min and the balance purge was 40 cc/min under the flowing nitrogen gas of 20 psi. Samples, in the weight range of 3–5 mg, were placed in a high temperature platinum pan and degraded as a function of temperature; their mass loss was recorded by the TGA Universal Software (TA Instruments, New Castle, DE 19720, USA).

Samples cut from filaments and printed coupons were mounted in epoxy and polished for microstructural analysis of the cross-sections with an optical microscope. Scanning electron microscopy (SEM) and field-emission (FE) SEM analyses were also done on the ABS w/5%CF and ABS w/CNT to analyze the distribution of the reinforcements (chopped fibers or nanotubes) and identify any impurities. Chopped carbon fibers and possible impurities were identified with Energy Dispersive Spectroscopy (EDS). Measurements of porosity within the ABS were conducted on the optical micrographs using ImageJ (National Institutes of Health public domain software).

For tensile testing, the strains were determined with an ARAMIS (Trilion Quality Systems, Plymouth Meeting, PA 19462, USA) photogrammetry system. The elastic modulus of materials was determined in two different manners: dynamically and mechanically. The dynamic testing consisted of impulse excitation in nominal conformance with ASTM C1259 [19]. Mechanical modulus was determined from the tensile data. Generally, dynamic and mechanical results were similar, and the dynamic testing ceased. Fracture toughness was determined according to ASTM D5045 [18], with exception of meeting some of the thickness or plasticity criteria; however, additional specimens of some materials tested with a greater thickness (10 mm) resulted in similar values, implying reasonable trends in the data and conclusions. Details of the material system, print layer height, coupon dimensions, Young’s modulus, ultimate strength, and fracture toughness for each tested coupon are displayed in the supplementary file (Tables S1–S3).

## 3. Results and Discussion

### 3.1. Thermal Stability

**Figure 1:**
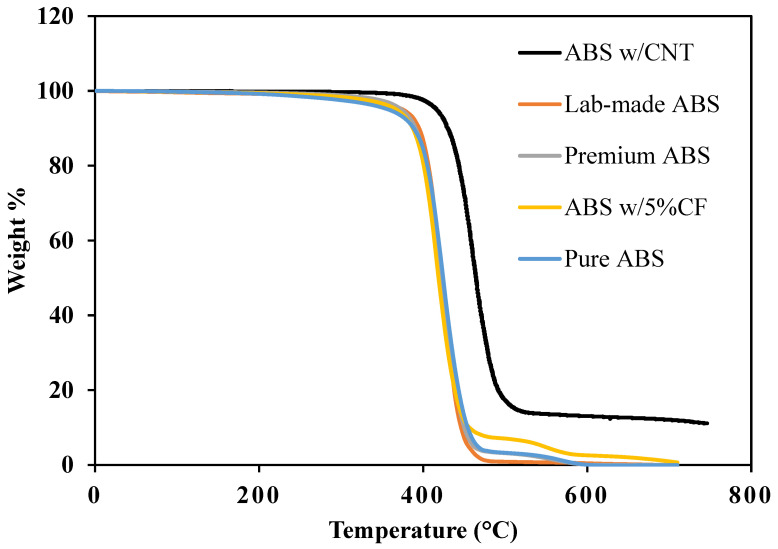
TGA Results of materials conducted in nitrogen gas.

The thermal stability of the various ABS filaments used in this study was investigated to characterize any difference between the source materials and to estimate the wt.% addition of either carbon-fiber or carbon nanotube reinforcements. Figure 1 shows the TGA (thermogravimetric analysis) results, performed in nitrogen gas. Premium ABS and Pure ABS are the only filaments to show a two-step degradation process. The onset of degradation occurs at approximately 300 °C, and continues until approximately 450 °C, indicative of the structural decomposition of the polymer matrix. The second window of thermal degradation, between 450 °C and 600 °C, is characteristic of residual combustion reactions. By 620 °C, only the ABS w/5%CF and ABS w/CNT partially remain. This two-step degradation is well understood and widely observed [20,21], but the data for the two specimens that did not degrade in these two steps needs to be understood.

Since the Lab-made ABS filament was produced from high purity ABS Filabot pellets by melt extrusion in a well-controlled environment, minimal contamination occurred, thereby resulting in little to no residuals after the 450 °C. Other studies that conducted TGA experiments on ABS also showed no residual combustions after 450 °C [22].

ABS w/5%CF exhibited both degradation steps mentioned above and a third small step between 600 °C and 700 °C. At the end of the second step, at 600 °C, approximately 3 wt.% of the specimen remained. This remaining material can be attributed to the chopped carbon fiber reinforcement. The amount of reinforcement is less than the advertised 5 wt.%. This discrepancy presents the necessity to characterize source material before designing structures, mainly if the structures are designed around the presence of reinforcements. Billah et al. obtained TGA data of 20 wt.% carbon fiber reinforced ABS. The carbon fiber in their material did not fully degrade by 700 °C, as expected. However, the carbon fiber reinforced ABS in this study underwent complete degradation. It is unclear why none of the ABS w/5%CF samples remained after reaching 700 °C.

ABS w/CNT exhibited the first degradation step, but at approximately 400 °C, 100 °C higher than all other filaments in this study. This is not unexpected, however. Kapoor et al. and Gutierrez et al. performed TGA experiments on ABS with multi-walled carbon nanotube reinforcement. Materials in both studies exhibited the onset of degradation at temperatures ranging from 380 to 400 °C [23,24]. The ABS w/CNT filament in this study did not fully degrade before reaching the max temperature of 745 °C. The data obtained by Kapoor et al. and Gutierrez et al. showed the same results. In their studies, the CNT wt.% present in the ABS filament was already known and agreed very well with the CNT wt.% remaining at the end of their TGA experiments. The remaining wt.% of the material in this study is 11%, and though 3DXTech did not disclose the amount of CNT reinforcement, we can conclude that this remaining 11 wt.% corresponds to the wt.% of CNT in the filament.

### 3.2. Microstructural Characterization

**Figure 2:**
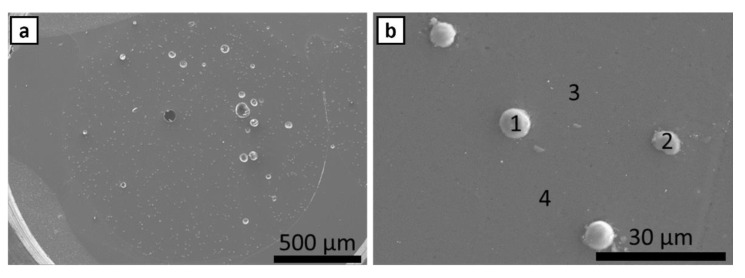
(**a**) FE SEM image of ABS w/5%CF. Numbered areas in (**b**) underwent EDS analysis.

**Figure 3:**
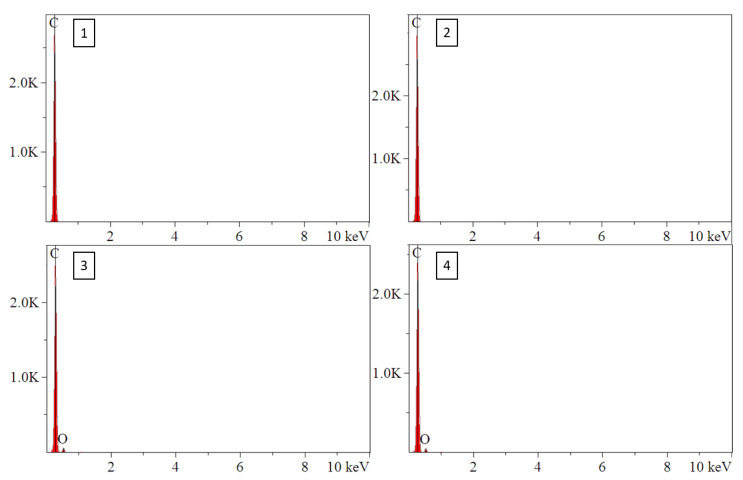
EDS spectra of ABS w/5%CF identifying chopped fibers and oxygen in ABS matrix. Numbers 1–4 correspond to the respective areas in Figure 2b.

**Figure 4:**
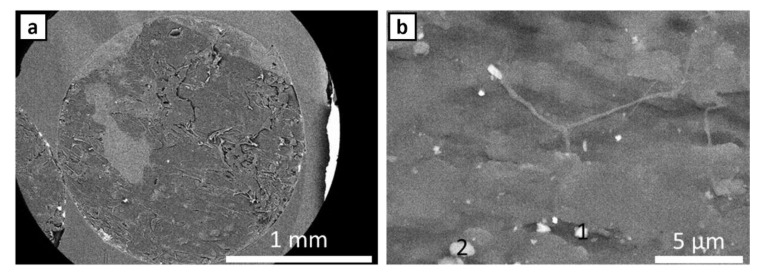
(**a**) FE SEM image of ABS w/CNT. Numbered areas in (**b**) underwent EDS analysis.

**Figure 5:**
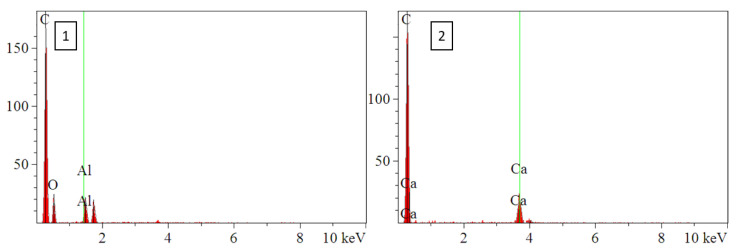
EDS spectra of ABS w/CNT showing presence of impurities. Numbers 1 and 2 correspond to the respective areas in Figure 4b.

FE SEM and EDS were performed on cross-sections of as supplied ABS filament for ABS w/5%CF and ABS w/CNT. SEM micrographs and the corresponding EDS signals Table 1 of ABS w/5%CF are shown in Figure 2 and Figure 3, respectively. Similarly, the micrographs and signals for ABS w/CNT are shown in Figure 4 and Figure 5. The areas marked as “1” and “2” in Figure 2b are cross-sectional views of the chopped carbon fiber present in the material. The filament pieces imaged in Figure 2 and Figure 4 were prepared from cuts made perpendicular to the length of the filament. Therefore, Figure 2b shows that the orientation of these fibers is parallel to the length of the filament. Figure 3 shows minimal oxygen in the matrix, but otherwise, the ABS w/5%CF filament is free of contaminants or impurities. However, this is not the case for ABS w/CNT filament. Small impurities of approximately 0.5–1 µm in diameter, shown as bright white spots in Figure 4b, can be seen in micrographs. These impurities may still negatively affect this material’s printing and, therefore, its overall mechanical performance. Figure 5 shows that these impurities are composed of carbon and oxygen, as expected, but also aluminum and calcium. Neither of those latter elements should be present in ABS nor carbon nanotubes. Aluminum and calcium are often used as catalysts in nanotube production and may be left over from fabrication processes. The mechanical properties sections below show that these impurities do not play a detrimental role in mechanical properties.

**Figure 6:**
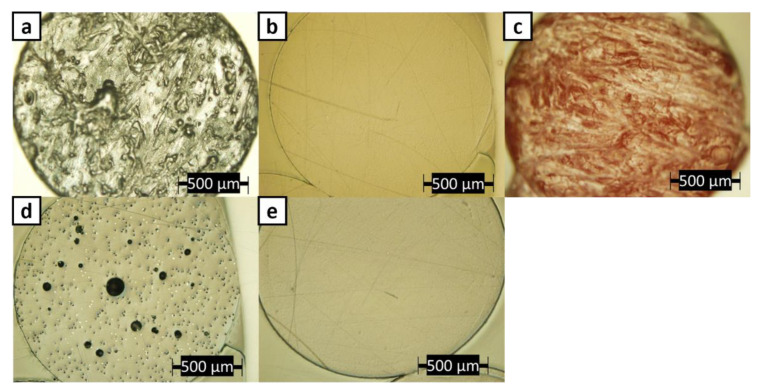
Optical images of cross-sections of (**a**) ABS w/CNT, (**b**) Lab-made ABS, (**c**) Premium ABS, (**d**) ABS w/5%CF, and (**e**) Pure ABS filaments.

Micrographs of a cross-section of each print filament material are shown in Figure 6. Before the printing of coupons, all ABS filament materials, except ABS w/5%CF, did not show significant porosity. In Figure 6d, the chopped carbon fiber appears as the white dots, while the porosity is identified by the large black circles and tiny black dots. Due to the size, placement, and distribution of the small black dots, it is concluded that they are holes from which chopped carbon fiber was removed. The chopped carbon fiber most likely was pulled-out from the ABS matrix during the sample preparation, whether by cutting or polishing the filament.

**Figure 7:**
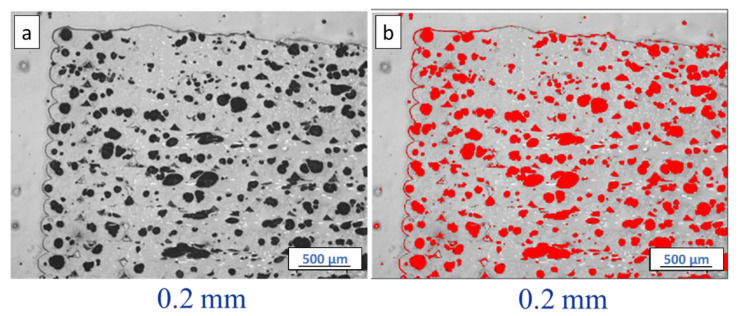
Example of identifying porosity in a printed sample with ImageJ software (ABS w/5%CF with 2 mm print layer height is shown). (**a**) is the as-taken optical image, and (**b**) shows the identified porosity in red.

**Figure 8:**
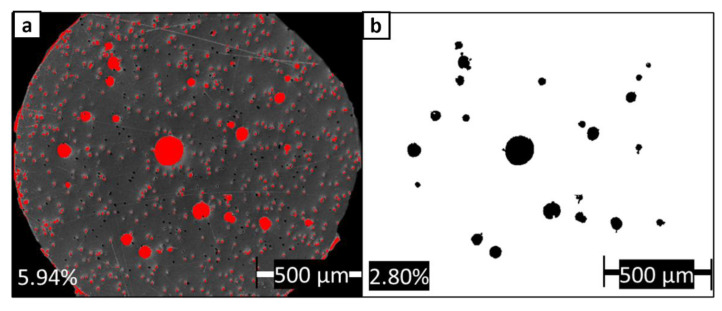
ImageJ analysis done on (**a**) porosity identified as large pores and holes left from removed chopped fibers, and (**b**) porosity identified only as large pores in ABS w/5%CF.

ImageJ software was used to estimate the amount of porosity observed in cross-sectional micrographs of ABS w/5%CF and Premium ABS. An example of the process is shown in Figure 7 for a 3-D printed coupon sample. Greyscale optical micrographs (Figure 7a) were split into color channels, and the image with the best contrast between material and pores was chosen. Black areas were identified as pores and marked in red (Figure 7b), and the percent area was calculated. Figure 8 shows the same micrograph from Figure 6d, but with the porosity identified and quantified. The black empty space outside the circular cross-section was removed from the area calculations. In Figure 8a, red symbolizes what can be observed as porosity, which includes both the large pores and the small holes left from the removed chopped fibers. When considering both of these artifacts as porosity, it is found that 5.94% of the filament area is porosity. Comparatively, in Figure 8b, only the large black circles are identified as porosity. When only using the large pores in the calculation, it is found that 2.80% of the filament area is porosity. Compared to the negligible porosity in the other filaments, ABS w/5%CF exhibits 2.80–5.94% porosity, which is a significant amount considering these images are taken before the filament is used for printing. The process of implanting chopped carbon fiber most likely introduced significant porosity to the filament. The porosity in the filament can detrimentally affect the printing of the ABS w/5%CF, as it may inhibit complete bonding between layers. This cascading effect of porosity forming is displayed in the micrographs of printed samples.

**Figure 9:**
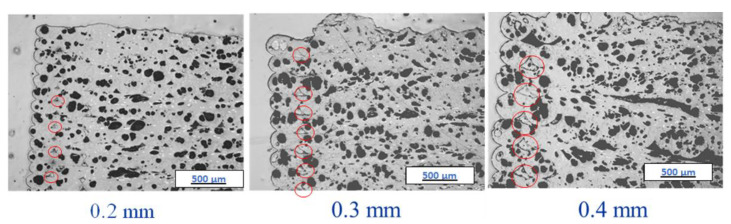
Microstructure and porosity of ABS w/5%CF as a function of layer height. Red circles mark some of the areas where epoxy has filled in a pore. Views at one of the corners for each printed sample are shown.

**Figure 10:**
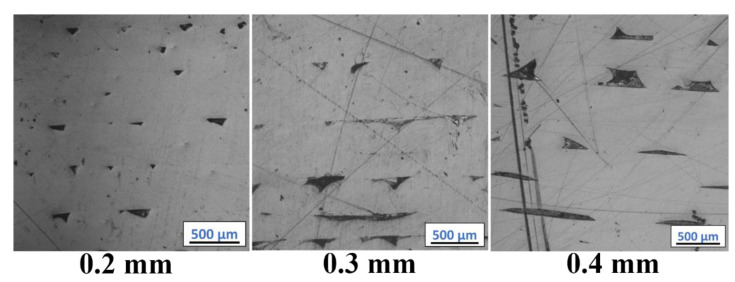
Microstructure and porosity of Premium ABS as a function of layer height. Views at the interior of the printed samples are shown.

Figure 9 and Figure 10, and corresponding Table 2 show the percent area of porosity observed with varying print layer heights in 3D printed ABS w/5%CF and Premium ABS, respectively. It is obvious from the images that the parts printed with the two different material systems exhibit differing amounts and patterns of porosity. In both materials, an increase in print layer height does relate to an increase in porosity, where ABS w/5%CF and Premium ABS range from 18.82–25.48% and 1.04–6.50% porosity, respectively. However, the porosity present in the ABS w/5%CF printed samples is higher than what is calculated with ImageJ. The red circles in Figure 9 show a pattern of pores formed at the beginning or ending of printed layers. These pores do not appear as black in the image because they were filled in by the epoxy used when mounting the sample for imaging. Figure 10 does not exhibit this pattern, as Premium ABS printed layers joined together with very few pores.

**Figure 11:**
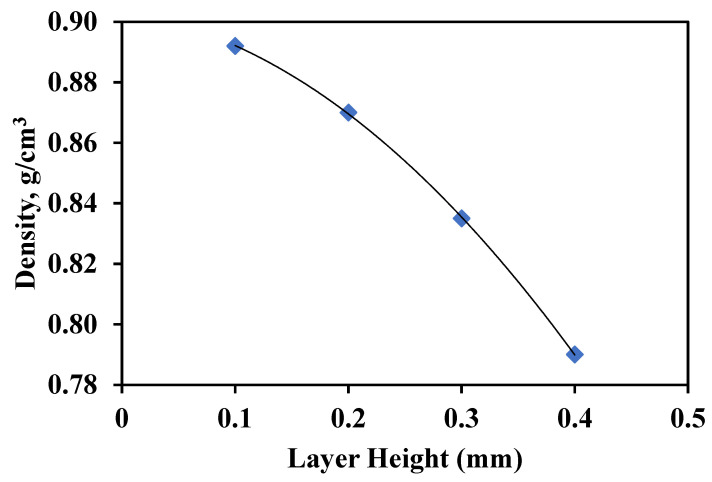
Density as a function of layer height for ABS w/5%CF.

**Figure 12:**
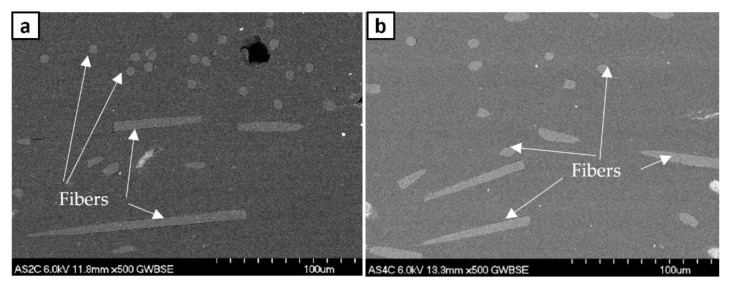
Scanning electron microscope images of carbon fiber distribution in (**a**) 0.2 mm and (**b**) 0.4 mm layer heights.

The density of ABS w/5%CF, shown in Figure 11, shows a decreasing trend as layer height increases. This data correlates with the observed increase in porosity. The carbon fiber distribution was relatively similar despite the porosity differences between layer heights, as shown in Figure 12. Fibers are printed horizontally relative to the print direction. Circular cross-sections and full lengths of fibers are both seen because alternating raster angles of 0° and 45° were used when printing. The printed sample displays a singular, preferred orientation of chopped fibers as was observed in the filament. The printing process did not affect the orientation of fibers; fibers were printed along the orientation of the print direction. The fibers were about 5–10 µm in diameter and 100 µm in length. Tekinalp et al. [15] observed a similar porosity pattern or orientation in their study where they varied the amount of chopped carbon fiber wt.% instead of the print layer height. In both cases, the increase in porosity may be due to shear separation of the fiber from the matrix during extrusion through the print head. Different extrusion methods can possibly solve these fabrication issues. The various print parameters can increase porosity in the final part and therefore require careful consideration before designing a 3D-printed structure.

**Figure 13:**
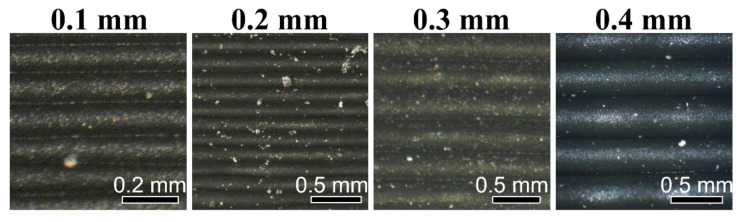
As-printed surfaces of Premium ABS as a function of layer height.

Figure 13 shows the as-printed surfaces of Premium ABS printed at different layer heights. As expected, parts printed with a lower layer height show more refined surfaces; however, print time is increased considerably for more refined parts. Superior fusion may allow larger layer height with low porosity; however, tolerances might be compromised. There is the possibility of varying other print parameters, such as nozzle or bed temperature, extrusion rate, etc., before the differences in porosity between the varying layer heights are negligible. When a negligible difference in density is obtained, faster print speeds can be applied for the presumably same mechanical properties.

### 3.3. Mechanical Properties

**Figure 14:**
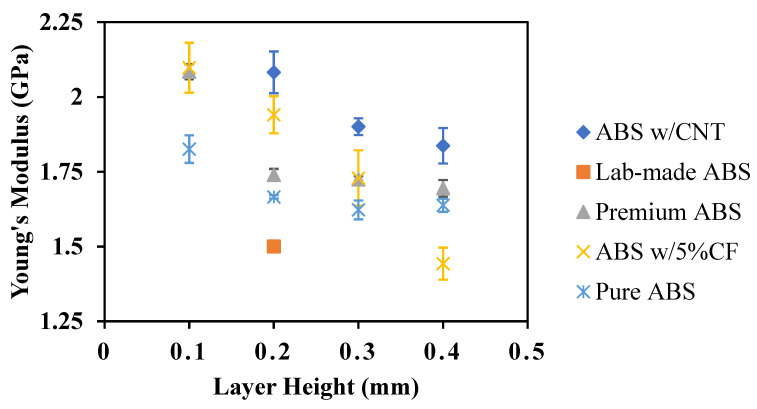
Young’s Modulus of ABS materials as a function of layer height.

**Figure 15:**
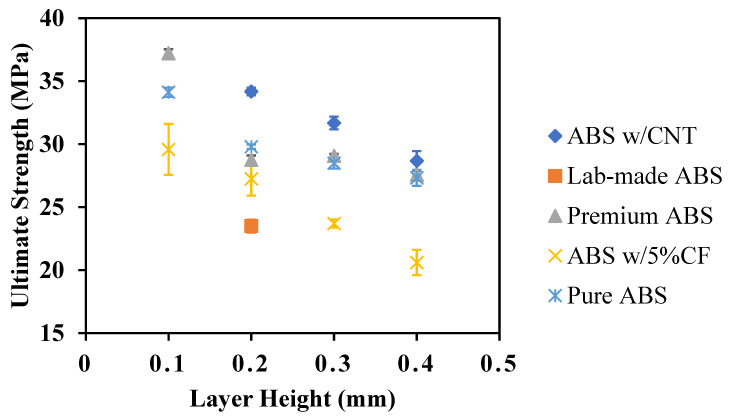
Ultimate strength of ABS materials as a function of layer height.

The average elastic modulus and ultimate strength for the various ABS polymers and print heights are shown in Figure 14 and Figure 15, respectively. Three coupons of each material were tested for each layer height. There is a general trend of decreased mechanical performance with increased print layer height. Premium ABS is substantially stronger than the other ABS coupons with no carbon additions.

**Figure 16:**
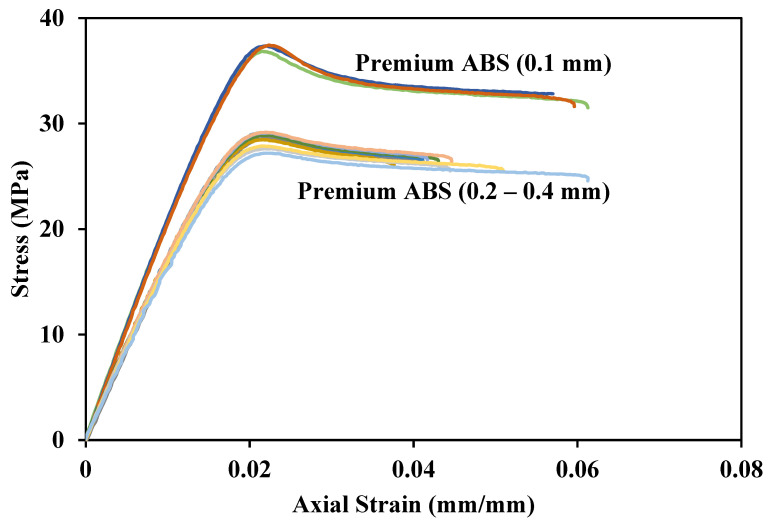
Tensile response of Premium ABS at all print layer heights.

**Figure 17:**
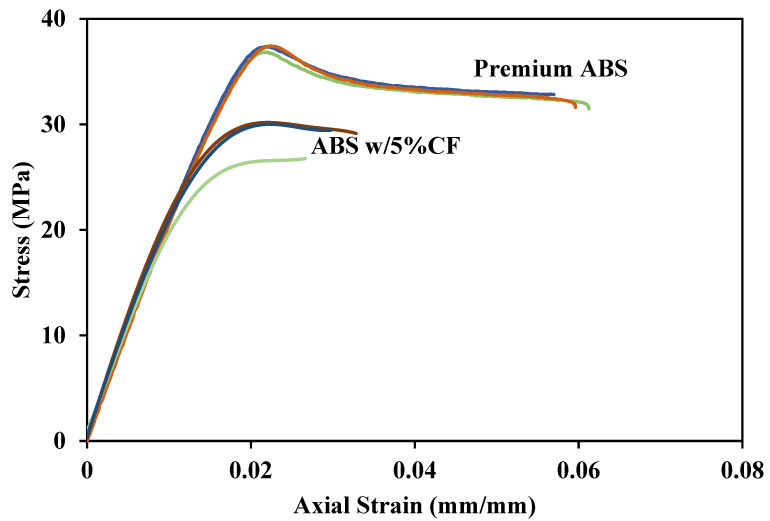
Tensile response of Premium ABS and ABS w/5%CF printed at a 0.1 mm layer height.

**Figure 18:**
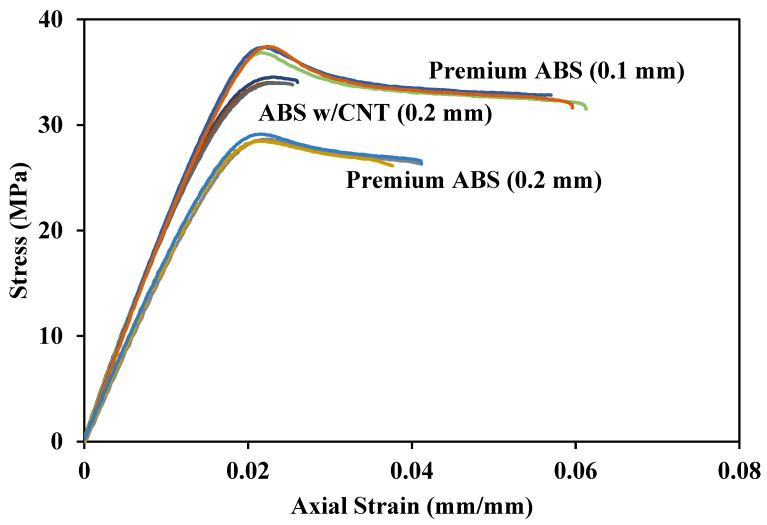
Tensile response of Premium ABS and ABS w/CNT printed at a 0.2 mm layer height.

**Figure 19:**
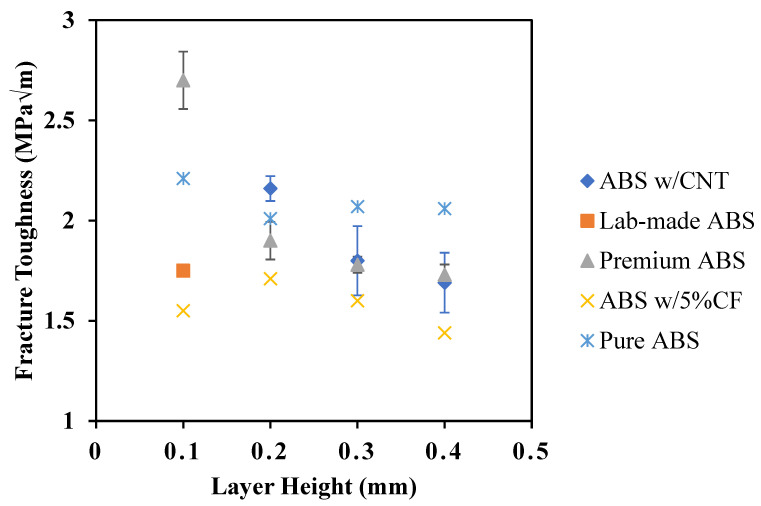
Fracture toughness as a function of layer height.

Figure 16, Figure 17, Figure 18 and Figure 19 show tensile responses of the ABS materials in this study. The different colored lines represent repeated testing to confirm the reproducibility of the results. Figure 16 compares the tensile response of Premium ABS printed at different layer heights. Premium ABS printed with a layer height of 0.1 mm exhibits a significantly stronger mechanical response than the same material printed at any other height, and this is consistent through multiple tests. When printed at a 0.1 mm height, the ultimate strength of Premium ABS is 28% higher than that of larger print heights. The drastic change of strength can be attributed to the increasing porosity and decreasing density of Premium ABS as it is printed at higher layer heights.

Figure 17 displays the tensile response of Premium ABS and ABS w/5%CF printed at a layer height of 0.1 mm. The two ABS material systems compared in Figure 17 have nearly identical elastic moduli, but ABS w/5%CF has a significantly lower ultimate strength. Repeated testing confirms that both material systems behaved consistently, but one ABS w/5%CF specimen underwent a premature fracture which is shown as the green line with the lowest ultimate strength. The low strength of the ABS w/5%CF material system is caused by excessive porosity present in the printed coupons, as shown in Figure 9. The incorporation of carbon reinforcement resulted in contaminants and porosity, weakening the material relative to Premium ABS. Due to the particle agglomerations and high porosity in the ABS w/5%CF, the independent effect of the reinforcing fibers could not be determined. Regardless of layer height, ABS w/5%CF is not a viable mechanical substitution for Premium ABS because of its suboptimal mechanical strength.

Figure 18 compares the tensile responses of Premium ABS and ABS w/CNT at their strongest layer heights. Challenges were encountered in printing the CNT material at the lowest layer height of 0.1 mm, and test coupons could not be obtained. ABS w/CNT with a print height of 0.2 mm exhibits an ultimate strength of 34 MPa, which is 19% higher than Premium ABS printed at the same height and only 8% lower than Premium ABS printed at a 0.1 mm height. These results are in good agreement with Thaler et al. [25]. They studied the mechanical properties of ABS with varying wt.% of CNT, from 0% to 10%, and found the ultimate strength to range from 30 MPa to 40 MPa, respectively. In their study, the highest Young’s modulus, 4000 MPa, was exhibited by ABS with 3 wt.% CNT. The increasing addition of the brittle CNT increased the stiffness of the host ABS material until a certain brittle-ductile threshold, which was found to be approximately 3 wt.%. In comparison, Podsiadly et al. also measured the mechanical behavior of 3D printed ABS, with a layer height of 0.2 mm and varying wt.% of CNT, from 0% to 10%, and found their ultimate strengths to widely range from 30 MPa, at 1 wt.%, to 100 MPa at 10 wt.% [26].

The increasing wt.%. of CNT, a high strength material, contributes to the composite material’s high ultimate strength, as is expected, but Podsiadly et al. did not report elongation behavior of ABS w/CNT [26]. It can be reasonably deduced that the composite material can withstand larger strains at lower wt.% of CNT additions, but as Thaler showed, CNT additions may actually increase the elastic modulus of ABS up to a certain point [25]. As determined by TGA, the CNT wt.% in this study’s filament is approximately 10%. The ultimate strength of ABS w/CNT printed with a 0.2 mm layer height, 34 MPa, is lower than that found by both Thaler et al. and Podsiadly et al. The lower strength can be attributed to the 0.2 mm layer height because, as the results in this study show, a smaller layer height of 0.1 mm usually exhibits higher ultimate strength. The elastic modulus of ABS w/CNT, approximately 2000 MPa, agrees with the results obtained by Thaler et al. The CNT wt.% of the material in this study is near 10 wt.%, which is 7 wt.% more than the determined brittle-ductile threshold, and therefor is expected to exhibit less stiffness. The significant amount of CNT reinforcement in the material in this study could explain why its observed Young’s modulus, 2000 MPa, is much lower than the 4000 MPa observed by Thaler et al. in their 3 wt.% CNT ABS material.

If print speed is essential, then ABS w/CNT printed at a 0.2 mm print height can structurally replace Premium ABS printed with a 0.1 mm print layer height. ABS w/CNT displays a higher elastic modulus and ultimate strength at each print height than any other material printed at the same height. It seems the minuscule impurities present in ABS w/CNT, as shown in Figure 4, did not detrimentally affect the printing of the material enough to weaken it substantially. If print parameters can be varied to correctly print ABS w/CNT at a 0.1 mm print layer height, it may display a higher strength than Premium ABS printed at the same height.

Figure 19 displays the average fracture toughness of all materials investigated in this study presented as a function of layer height. Fracture toughness was calculated with Equation 1, where $P_{Q}$. is the applied load, B. is the thickness of the specimen, a. is the crack length, and W. is the width of the specimen (ASTM D5045) [18].

(1) $$K_{Q}=\left(\frac{6P_{Q}\sqrt{a}}{BW}\right)\frac{1.99-\frac{a}{W}\left(1-\frac{a}{W}\right)\left(2.15-3.93\frac{a}{W}+2.7{\left(\frac{a}{W}\right)}^{2}\right)}{\left(1+2\frac{a}{W}\right){\left(1-\frac{a}{W}\right)}^{\frac{3}{2}}}$$

Similar to tensile mechanical properties, fracture toughness decreases with increasing layer height. However, the trends are less pronounced with ABS w/CNT and Premium ABS producing the highest fracture toughness values for ABS systems printed with a 0.2 mm layer height. At larger print heights, Pure ABS outperforms all other materials. There is slight disagreement with the trend in the fracture toughness of ABS w/5%CF, where a 0.2 mm print layer height results in a higher fracture toughness than a 0.1 mm print layer height. However, fracture toughness of ABS w/5%CF or all layer heights exhibit a small range of 0.27 MPa√m (from 1.44 to 1.71 MPa√m). The significant porosity present in the ABS w/5%CF specimen is most likely the cause for low fracture toughness values, as it was for the low tensile strength values. Premium ABS with 0.1 mm layer height exhibits higher fracture toughness than all other ABS material and print height combinations; its fracture toughness of 2.7 MPa√m is 22% higher than the next highest value (2.2 MPa√m).

However, similar to the tensile tests, if ABS w/CNT can be adequately printed with a print layer height of 0.1 mm, its fracture toughness may outperform Premium ABS printed at the same print layer height. ABS w/CNT exhibits a higher or similar fracture toughness to Premium ABS at any given print layer height, further affirming the use of ABS w/CNT as a structural replacement to Premium ABS. There is a possibility for a higher print layer height and faster print speed with ABS w/CNT. It is also interesting to note that Pure ABS exhibits the most consistent fracture toughness, where the difference in fracture toughness may be negligible between the print layer heights. Combined with ultimate strength, however, it is shown that a print layer height of 0.4 mm cannot be substituted for 0.2 mm while maintaining the same structural integrity.

**Figure 20:**
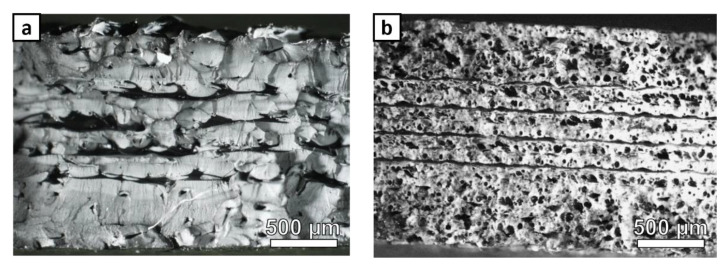
Fracture surfaces of tensile tested specimens with a 0.2 mm layer height: (**a**) Pure ABS and (**b**) ABS w/5%CF.

Example fracture surfaces for Pure ABS and ABS w/5%CF with 0.2 mm layer height are shown in Figure 20. Within fracture surfaces for both materials, separation of the layers in the center of the samples occurs, indicating weak fusion between print layers. In contrast, the print layers remain fused at the edges of one another. The Pure ABS specimen has some porosity between layers and very little porosity within the layers. The ABS w/5%CF has significant porosity within the print layers, which appears to become interconnected during tensile testing. The ultimate strength of ABS w/5%CF with 0.2 mm print layer heights (1.71 MPa√m) was lower than all other coupons printed with a 0.2 mm print height, except the Lab-made ABS coupon. In general, porosity between layers and within the layer affects the strength of the 3D-printed material systems, but porosity between the layers seems to have a more adverse effect.

ABS w/CNT, Premium ABS, ABS w/5%CF, and Pure ABS were all sourced from the same manufacturer yet varied considerably in mechanical strength. The carbon nanotube reinforcements help increase the ABS system’s elastic modulus, ultimate strength, and fracture toughness, regardless of print height. However, Premium ABS is labeled “higher-grade” relative to Pure ABS. Premium ABS does not contain any reinforcements or modifications, yet it outperforms all ABS systems except ABS w/CNT. The “grade” of the ABS polymer present in the ABS w/CNT material system was not identified, which confounds comparisons between it and Premium ABS. It is also possible that the print parameters chosen for this study, which remained constant through all material systems, were ideal for Premium ABS and not for Pure ABS. One beneficial aspect of pure ABS is the consistent fracture toughness of over 2 MPa√m at any print height.

ABS polymer printed at a 0.1 mm layer height will exhibit a better consolidation, higher density, and lower porosity, but will require a longer print time. The same part can be printed much faster with larger layer heights but with sacrificed mechanical properties with the same overall dimensions. In most cases, ABS w/CNT is mechanically similar in strength or stronger than Premium ABS. The addition of CNT reinforcement can allow for larger print layer heights, faster print speeds, and a more efficient manufacturing process. Not all reinforcements, such as chopped carbon fiber, will necessarily increase mechanical strength. Characterizing the material systems and studying their mechanical properties will help aid design choices based on whether they prioritize mechanical strength or print speed.

## 4. Conclusions

Not all ABS filament is made equally. FE SEM illuminated the porosity and impurities in the as-supplied filaments of ABS-carbon composites. Thermal stability measurements show the inconsistencies in temperatures at which the ABS polymer matrix decomposes, residual combustion reactions begin, and even the temperature at which the material has completed degradation. With the gain of printing speed comes the detriment of porosity. Porosity increased in Premium ABS and ABS w/5%CF when print layer height was increased. The presence of large porosity in the ABS w/5%CF filament further exacerbated the increase in porosity and prevented proper fusion of ABS polymer between print layers. Both materials exhibited the least porosity when printed with a 0.1 mm print layer height, but the difference in porosity between the material systems is significant. When printed at a 0.1 mm print height, Premium ABS and ABS w/5%CF exhibit 1.04% and 18.82% porosity. The effect of the porosity was also visible on the surface of Pure ABS, with a less refined structure at larger print heights.

The increasing porosity and decreasing density caused a general trend of decreasing strength and modulus with increasing layer height for ABS and ABS with various additives. The type and quality of ABS materials strongly influence mechanical properties as well, with premium grade giving better fracture toughness and strength than pure ABS. ABS exhibited the highest strength and elastic modulus with carbon nanotube additions. In all cases, ABS w/CNT performed as well as or better than Premium ABS and consistently better than Pure ABS. However, that is not the case for ABS w/5%CF. The large porosity in ABS w/5%CF prevents it from being mechanically and structurally comparably to Premium ABS or even Pure ABS. Regardless of the quality or grade of the ABS filament, the addition of chopped carbon fiber did provide mechanical benefits. If print speed is crucial, Pure and Premium ABS can often be replaced with ABS w/CNT to allow larger print layer heights. The optimization of printing parameters can be conducted for a given polymer and printer to increase the functionality of different materials, such as by lowering the porosity.

## Figures and Tables

**Table 1:** Tabulated EDS spectra data corresponding to Figure 2b and Figure 4b.

Figure	Element	Line	Intensity	Conc	Mole Conc
			c/s	wt.%	wt.%
Figure 2b-1	C	Ka	407.4	100.0	100.0
Figure 2b-2	C	Ka	434.6	100.0	100.0
Figure 2b-3	C	Ka	372.5	93.9	95.4
	O	Ka	8.5	6.1	4.6
Figure 2b-4	C	Ka	363.9	94.7	96.0
	O	Ka	7.1	5.3	4.0
Figure 4b-1	C	Ka	28.6	66.6	78.3
	O	Ka	3.3	12.4	11.0
	Al	Ka	4.1	10.6	5.5
	Si	Ka	3.3	10.4	5.2
Figure 4b-2	C	Ka	29.2	32.9	62.0
	Ca	Ka	5.3	67.1	38.0

**Table 2:** Porosity percent area determinations of optical images in Figure 9 and Figure 10.

Material	Layer Height (mm)	Porosity Perecnt Area (%)
ABS w/5%CF	0.2	18.8
ABS w/5%CF	0.3	22.0
ABS w/5%CF	0.4	25.5
Premium ABS	0.2	1.0
Premium ABS	0.3	2.8
Premium ABS	0.4	6.5

## Data Availability

Not applicable.

## Supplementary Materials

The following are available online at https://www.mdpi.com/article/10.3390/polym14102105/s1, Table S1: Average Young’s modulus and ultimate strength for each material and layer height, Table S2: Dimensions, Young’s modulus, and ultimate strength for all test coupons, Table S3: Average fracture toughness values obtained for each material and layer height.
