# Supplementary material for: Effect of Reinforcements and 3-D Printing Parameters on the Microstructure and Mechanical Properties of Acrylonitrile Butadiene Styrene (ABS) Polymer Composites
Source: Polymers (Basel). 2022 May 21;14(10):2105. doi: 10.3390/polym14102105 (PMC9145829; doi:10.3390/polym14102105)
Supplement: Supplementary file 1 [file polymers-14-02105-s001.zip › polymers-1702267-supplementary.pdf]

Effect of Reinforcements and 3-D Printing Parameters on the Microstructure and Mechanical Properties of Acrylonitrile Butadiene Styrene (ABS) Polymer Composites

Ved S. Vakharia [1], Mrityunjay Singh [2,*], Anton Salem [3], Michael C. Halbig [4,*] and Jonathan A. Salem [4]

1 NASA Pathway Intern, Department of Mechanical and Aerospace Engineering, University of California, San Diego, La Jolla, CA 92092, USA; ved.vakharia@gmail.com
2 Ohio Aerospace Institute, Cleveland, OH 44142, USA
3 NASA Intern currently at VulcanForms, Inc., Burlington, MA 01803, USA; antonsalem1@gmail.com
4 NASA Glenn Research Center, Cleveland, OH 44135, USA; jonathan.a.salem@nasa.gov
* Correspondence: mrityunjaysingh@oai.org (M.S.); michael.c.halbig@nasa.gov (M.C.H.)

Supplementary Materials

Table S1. Average Young's modulus and ultimate strength for each material and layer height.

Material	Layer Height (mm)	Young's Modulus (MPa)	Ultimate Strength (MPa)
ABS w/CNT	0.2	2082	34.2
ABS w/CNT	0.3	1901	31.7
ABS w/CNT	0.4	1837	28.7
Lab-made ABS	0.2	1500	23.5
Premium ABS	0.1	2084	37.2
Premium ABS	0.2	1739	28.8
Premium ABS	0.3	1724	29.1
Premium ABS	0.4	1695	27.6
ABS w/5%CF	0.1	2098	29.6
ABS w/5%CF	0.2	1941	27.3
ABS w/5%CF	0.3	1729	23.7
ABS w/5%CF	0.4	1443	20.6
Pure ABS	0.1	1826	34.1
Pure ABS	0.2	1666	29.8
Pure ABS	0.3	1622	28.5
Pure ABS	0.4	1638	27.3

Table S2. Dimensions, Young's modulus, and ultimate strength for all test coupons.

Material	Specimen	Layer Height (mm)	Width (in)	Thickness (in)	Area (in^2)	Young's Modulus (MPa)	Ultimate Strength (MPa)
ABS w/CNT	02_ABS-CNT_1	0.2	0.524	0.123	0.064	2160.66	34.53
ABS w/CNT	02_ABS-CNT_2	0.2	0.528	0.124	0.065	2059.62	34.01
ABS w/CNT	02_ABS-CNT_3	0.2	0.529	0.124	0.066	2026.79	33.98
ABS w/CNT	03_ABS-CNT_1	0.3	0.530	0.131	0.069	1892.67	31.11
ABS w/CNT	03_ABS-CNT_2	0.3	0.531	0.131	0.069	1877.61	31.85
ABS w/CNT	03_ABS-CNT_3	0.3	0.527	0.130	0.069	1931.64	32.09
ABS w/CNT	04_ABS-CNT_1	0.4	0.523	0.129	0.067	1868.37	29.53
ABS w/CNT	04_ABS-CNT_2	0.4	0.531	0.133	0.071	1768.47	28.01
ABS w/CNT	04_ABS-CNT_3	0.4	0.527	0.132	0.069	1874.13	28.47
Lab-made ABS	02_ABS-HM_1	0.2	0.520	0.113	0.059	1503.34	23.86
Lab-made ABS	02_ABS-HM_2	0.2	0.513	0.118	0.061	1496.57	23.14
Premium ABS	01_ABS-P_1	0.1	0.531	0.128	0.068	2085.79	36.85
Premium ABS	01_ABS-P_2	0.1	0.530	0.126	0.066	2108.96	37.36

Premium ABS	01_ABS-P_3	0.1	0.527	0.129	0.068	2058.40	37.43
Premium ABS	02_ABS-P_1	0.2	0.529	0.139	0.073	1716.34	28.64
Premium ABS	02_ABS-P_2	0.2	0.528	0.138	0.073	1752.64	28.47
Premium ABS	02_ABS-P_3	0.2	0.529	0.137	0.072	1749.30	29.14
Premium ABS	03_ABS-P_1	0.3	0.534	0.141	0.075	1715.71	28.90
Premium ABS	03_ABS-P_2	0.3	0.529	0.141	0.074	1721.32	29.13
Premium ABS	03_ABS-P_3	0.3	0.523	0.142	0.074	1735.86	29.18
Premium ABS	04_ABS-P_1	0.4	0.523	0.140	0.073	1716.05	27.62
Premium ABS	04_ABS-P_2	0.4	0.523	0.140	0.073	1704.58	27.87
Premium ABS	04_ABS-P_3	0.4	0.529	0.141	0.075	1663.00	27.20
ABS w/5%CF	01_ABS-CF_1	0.1	0.5105	0.1205	0.062	2029.39	27.25
ABS w/5%CF	01_ABS-CF_2	0.1	0.5125	0.116	0.059	2190.80	30.92
ABS w/5%CF	01_ABS-CF_3	0.1	0.5145	0.1195	0.061	2073.19	30.55
ABS w/5%CF	02_ABS-CF_1	0.2	0.519	0.1253	0.065	1938.52	26.51
ABS w/5%CF	02_ABS-CF_2	0.2	0.515	0.1245	0.064	1879.75	26.46
ABS w/5%CF	02_ABS-CF_3	0.2	0.519	0.123	0.064	2003.65	28.79
ABS w/5%CF	03_ABS-CF_1	0.3	0.525	0.1325	0.070	1792.51	23.33
ABS w/5%CF	03_ABS-CF_2	0.3	0.5195	0.13	0.068	1622.56	23.93
ABS w/5%CF	03_ABS-CF_3	0.3	0.519	0.135	0.070	1771.56	23.83
ABS w/5%CF	04_ABS-CF_1	0.4	0.527	0.1335	0.070	1397.14	20.48
ABS w/5%CF	04_ABS-CF_2	0.4	0.5245	0.135	0.071	1429.49	19.68
ABS w/5%CF	04_ABS-CF_3	0.4	0.525	0.132	0.069	1502.05	21.67
Pure ABS	01_ABS_1	0.1	0.5205	0.1305	0.068	1778.28	34.10
Pure ABS	01_ABS_2	0.1	0.522	0.1345	0.070	1828.91	33.76
Pure ABS	01_ABS_3	0.1	0.522	0.129	0.067	1870.49	34.50
Pure ABS	02_ABS_1	0.2	0.534	0.1395	0.074	1664.03	29.92
Pure ABS	02_ABS_2	0.2	0.5285	0.139	0.073	1661.66	29.60
Pure ABS	02_ABS_3	0.2	0.531	0.146	0.078	1672.14	29.88
Pure ABS	03_ABS_1	0.3	0.5301	0.153	0.081	1588.29	28.23
Pure ABS	03_ABS_2	0.3	0.53	0.148	0.078	1650.16	29.01
Pure ABS	03_ABS_3	0.3	0.53	0.151	0.080	1628.49	28.27
Pure ABS	04_ABS_1	0.4	0.5325	0.148	0.079	1639.13	27.17
Pure ABS	04_ABS_2	0.4	0.526	0.149	0.078	1615.24	28.01
Pure ABS	04_ABS_3	0.4	0.5295	0.151	0.080	1659.40	26.78

Table S3. Average fracture toughness values obtained for each material and layer height.

Material	Layer Height (mm)	Fracture Toughness (MPa√m)
ABS w/CNT	0.2	2.16
ABS w/CNT	0.3	1.80
ABS w/CNT	0.4	1.69
Lab-made ABS	0.1	1.75
Premium ABS	0.1	2.70
Premium ABS	0.2	1.90
Premium ABS	0.3	1.78
Premium ABS	0.4	1.73
ABS w/5%CF	0.1	1.55
ABS w/5%CF	0.2	1.71
ABS w/5%CF	0.3	1.60
ABS w/5%CF	0.4	1.44
Pure ABS	0.1	2.21
Pure ABS	0.2	2.01
Pure ABS	0.3	2.07

Pure ABS	0.4	2.06
